# Supplementary material for: Machine learning of pair-contact process with diffusion
Source: Sci Rep. 2022 Nov 17;12:19728. doi: 10.1038/s41598-022-23350-2 (PMC9672110; doi:10.1038/s41598-022-23350-2)
Supplement: Supplementary file 1 — Supplementary Information. [file 41598_2022_23350_MOESM1_ESM.pdf]

Machine learning of pair-contact process with diffusion

Jianmin Shen[1], Wei Li[1,2,*], Shengfeng Deng[3], Dian Xu[1], Shiyang Chen[1], and Feiyi Liu[1,4]

[1]Key Laboratory of Quark and Lepton Physics (MOE) and Institute of Particle Physics, Central China Normal University, Wuhan 430079, China
[2]Max-Planck-Institute for Mathematics in the Sciences, 04103 Leipzig, Germany
[3]Institute of Technical Physics and Materials Science, Center for Energy Research, Budapest 1121, Hungary
[4]Institute for Physics,Eötvös Loránd University, 1/A Pázmány P. Sétány, H-1117, Budapest, Hungary
[*]liw@mail.ccnu.edu.cn

ABSTRACT

This is the supplementary material for the article “Machine learning of pair-contact process with diffusion”. One can get the raw data and algorithms in the manuscript by using the following codes.

PCPD’s data sets for training and test

We can apply the following Python code to generate configurations for machine learning.

```
import numpy as np
import random
import matplotlib.pyplot as plt

# -----------generate-------------------------------------------
def Update(V_pro, dl, dr, ann, cl):
    L = np.shape(V_pro)[0]
    V_aft = np.copy(V_pro)
    for i in range(L):
        h = (i - 1 + L) % L
        j = (i + 1) % L
        k = (i + 2) % L
        l = (i + 3) % L

        prob = random.random()
        if prob<dl: #diff to right
            if V_pro[j] == 1 and  V_pro[k] ==0:
                V_aft[j] =0
                V_aft[k] =1

        elif prob<dr: #diff to left
            if V_pro[j] == 1 and  V_pro[i] ==0:
                V_aft[j] =0
                V_aft[i] =1

        elif prob < ann:
            if V_pro[j] ==1 and V_pro[k] ==1:
                V_aft[j] =0
                V_aft[k] =0

        elif prob < cl: #create at right
            if V_pro[j] ==1 and V_pro[k] ==1 and V_pro[l] ==0:
                V_aft[l] =1

        else: #create at left
            if V_pro[i] ==1 and V_pro[j] ==1 and V_pro[h] ==0:
                V_aft[h] =1
    return V_aft
```

```python
ncyc_train = 2500
ncyc_test = 500

# ptrain = [0.1 + x*0.1 for x in range(10)]
# ptrain = [0.1,0.2,0.3,0.4,0.5,0.6,0.7,0.8,0.9,1.0]
ptrain = [0.0375 + x*0.0025 for x in range(31)]
# print(ptrain)
# ptrain = [0.0 + x*0.025 for x in range(41)]
# ptrain = [0.1, 0.9]
ptest = ptrain

d = 0.0
pc = 0.077092
# L = 1000
# T = 1000

dl = d/2
dr = d


# -------------------------------------------------------
z = 1.

LENGTH_GET = [80]
STEP = [[1,81]]
for size in range(len(LENGTH_GET)):
    # Size
    LENGTH = LENGTH_GET[size]
    # Time
    TIME = np.math.ceil(LENGTH**z)
    a_test = []
    for p in ptest:
        ann = dr + p*(1-dr)
        cl = ann + (1-p)*(1-dr)/2
        for icyc in range(0, ncyc_test):
            # vector = []
            vector_LT = []
            V_pro = np.ones(LENGTH)
            vector_LT.append(V_pro)
            for i in range(TIME):
                V_pro = Update(V_pro, dl, dr, ann, cl)
                vector_LT.append(V_pro)
                # print(vector_LT)
            # if icyc == 99:
            #     plt.imshow(vector_LT)
            #     plt.show()
            # if icyc ==1800:
            #     print(vector_LT)
            a_test.append(vector_LT[STEP[size][0] - 1:STEP[size][1] - 1])
    # print(a_test)

    np.save('./data/' + 'xtest_' + str(LENGTH) + '_' + str(TIME) + '.npy',
            a_test)

    fy = open('./data/' + 'ytest_' + str(LENGTH) + '_' + str(TIME) + '.dat',
              "w")
    for p in ptest:
        for icyc in range(0, ncyc_test):
            if (p < pc):
                fy.write("%5d \n" % 1)
            else:
                fy.write("%5d \n" % 0)
    fy.close()

    b_test = []
    for p in ptrain:
        ann = dr + p*(1-dr)
        cl = ann + (1-p)*(1-dr)/2
        for icyc in range(0, ncyc_train):
            # vector = []
```

```
            vector_LT = []
            V_pro = np.ones(LENGTH)
            vector_LT.append(V_pro)
            for i in range(TIME):
                V_pro = Update(V_pro, dl, dr, ann, cl)
                vector_LT.append(V_pro)

            b_test.append(vector_LT[STEP[size][0] - 1:STEP[size][1] - 1])

    np.save('./data/' + 'xtrain_' + str(LENGTH) + '_' + str(TIME) + '.npy',
            b_test)

    fy = open('./data/' + 'ytrain_' + str(LENGTH) + '_' + str(TIME) + '.dat',
              "w")
    for p in ptrain:
        for icyc in range(0, ncyc_train):
            if (p < pc):
                fy.write("%5d \n" % 1)
            else:
                fy.write("%5d \n" % 0)
    fy.close()
```

Machine learning algorithms

The following code is for helping importing data.

```
"""Functions for downloading and reading MNIST data."""
import gzip
import os
import urllib
import numpy
SOURCE_URL = 'http://yann.lecun.com/exdb/mnist/'


def maybe_download(filename, work_directory):
    filepath = os.path.join(work_directory, filename)
    return filepath


def _read32(bytestream):
    dt = numpy.dtype(numpy.uint32).newbyteorder('>')
    return numpy.frombuffer(bytestream.read(4), dtype=dt)


def extract_images(filename, lx, ly):
    """Extract the images into a 4D uint8 numpy array [index, y, x, depth]."""
    print('Extracting', filename, 'aaaaaa')

    #with gzip.open(filename) as bytestream:
    #    magic = _read32(bytestream)
    #    if magic != 2051:
    #        raise ValueError(
    #            'Invalid magic number %d in MNIST image file: %s' %
    #            (magic, filename))
    #    num_images = _read32(bytestream)
    #    rows = _read32(bytestream)
    #    cols = _read32(bytestream)
    #    buf = bytestream.read(rows * cols * num_images)
    #    data = numpy.frombuffer(buf, dtype=numpy.uint8)
    #    data = data.reshape(num_images, rows, cols, 1)
    data = numpy.load(filename)
    dim = data.shape[0]
    print('xxxxxxxxxxxxxxxxxxx')
    data = data.reshape(dim, lx, ly, 1)
    print(data.shape)
    return data


def dense_to_one_hot(labels_dense, num_classes=10):
```

```
    """Convert class labels from scalars to one-hot vectors."""
    num_labels = labels_dense.shape[0]
    index_offset = numpy.arange(num_labels) * num_classes
    labels_one_hot = numpy.zeros((num_labels, num_classes))
    labels_one_hot.flat[index_offset + labels_dense.ravel()] = 1
    return labels_one_hot


def extract_labels(nlabels, filename, one_hot=False):
    """Extract the labels into a 1D uint8 numpy array [index]."""
    print('Extracting', filename, 'bbbccicicicicib')

    labels = numpy.loadtxt(filename, dtype='uint8')

    if one_hot:
        print("LABELS ONE HOT")
        print(labels.shape)
        XXX = dense_to_one_hot(labels, nlabels)
        print(XXX.shape)
        return dense_to_one_hot(labels, nlabels)
    print("LABELS")
    print(labels.shape)
    return labels


class DataSet(object):
    def __init__(self, images, labels, fake_data=False):
        if fake_data:
            self._num_examples = 80000000
        else:
            assert images.shape[0] == labels.shape[0], (
                "images.shape: %s labels.shape: %s" %
                (images.shape, labels.shape))
            self._num_examples = images.shape[0]
            # Convert shape from [num examples, rows, columns, depth]
            # to [num examples, rows*columns] (assuming depth == 1)
            assert images.shape[3] == 1
            images = images.reshape(images.shape[0],
                                    images.shape[1] * images.shape[2])
            # Convert from [0, 255] -> [0.0, 1.0].
            images = images.astype(numpy.float32)
            # images = numpy.multiply(images, 1.0 / 255.0) # commented since it is ising variables
            images = numpy.multiply(images, 1.0)  # multiply by one, instead
        self._images = images
        self._labels = labels
        self._epochs_completed = 0
        self._index_in_epoch = 0

    @property
    def images(self):
        return self._images

    @property
    def labels(self):
        return self._labels

    @property
    def num_examples(self):
        return self._num_examples

    @property
    def epochs_completed(self):
        return self._epochs_completed

    def next_batch(self, batch_size, fake_data=False):
        """Return the next `batch_size` examples from this data set."""
        if fake_data:
            fake_image = [1.0 for _ in xrange(784)]
            fake_label = 0
            return [fake_image for _ in xrange(batch_size)
```

```
                    ], [fake_label for _ in xrange(batch_size)]
        start = self._index_in_epoch
        self._index_in_epoch += batch_size
        if self._index_in_epoch > self._num_examples:
            # Finished epoch
            self._epochs_completed += 1
            # Shuffle the data
            perm = numpy.arange(self._num_examples)
            numpy.random.shuffle(perm)
            self._images = self._images[perm]
            self._labels = self._labels[perm]
            # Start next epoch
            start = 0
            self._index_in_epoch = batch_size
            assert batch_size <= self._num_examples
        end = self._index_in_epoch
        return self._images[start:end], self._labels[start:end]


def read_data_sets(nlabels, lx, ly, train_dir, fake_data=False, one_hot=False):
    class DataSets(object):
        pass

    data_sets = DataSets()
    if fake_data:
        data_sets.train = DataSet([], [], fake_data=True)
        data_sets.validation = DataSet([], [], fake_data=True)
        data_sets.test = DataSet([], [], fake_data=True)
        return data_sets

    z = 1.
    ly_1 = numpy.math.ceil(lx**z)
    TRAIN_IMAGES = 'xtrain_' + str(lx) + '_' + str(ly_1) + '.npy'
    TRAIN_LABELS = 'ytrain_' + str(lx) + '_' + str(ly_1) + '.dat'
    TEST_IMAGES = 'xtest_' + str(lx) + '_' + str(ly_1) + '.npy'
    TEST_LABELS = 'ytest_' + str(lx) + '_' + str(ly_1) + '.dat'
    VALIDATION_SIZE = 0
    local_file = maybe_download(TRAIN_IMAGES, train_dir)
    train_images = extract_images(local_file, lx, ly)
    print('after train images xxxxxxxxxxxxxxxxxxxxxxxxxxxxxxxxxxxxxxxxxxxx')
    local_file = maybe_download(TRAIN_LABELS, train_dir)
    train_labels = extract_labels(nlabels, local_file, one_hot=one_hot)

    local_file = maybe_download(TEST_IMAGES, train_dir)
    test_images = extract_images(local_file, lx, ly)
    local_file = maybe_download(TEST_LABELS, train_dir)
    test_labels = extract_labels(nlabels, local_file, one_hot=one_hot)
    validation_images = train_images[:VALIDATION_SIZE]
    validation_labels = train_labels[:VALIDATION_SIZE]
    train_images = train_images[VALIDATION_SIZE:]
    train_labels = train_labels[VALIDATION_SIZE:]
    data_sets.train = DataSet(train_images, train_labels)
    data_sets.validation = DataSet(validation_images, validation_labels)
    data_sets.test = DataSet(test_images, test_labels)
    return data_sets
```

The FCN algorithm

The PCPD's supervised machine learning algorithm by fully connected neural network is as follows:

```
import tensorflow as tf
import input_data
import sys
import numpy as np
import time
start = time.time()

numberlabels = 2
hiddenunits1 = 100
lamb = 0.01  # regularization parameter
```

```
batchsize_test = 10000
learning_rate = 0.0001
batch_size = 1024
trainstep = 20000

def weight_variable(shape):
    initial = tf.truncated_normal(
        shape, stddev=0.01)
    return tf.Variable(initial)

def bias_variable(shape):
    initial = tf.constant(
        0.01, shape=shape)  #shape=[2]     [[0.01 0.01] [0.01 0.01]]
    return tf.Variable(initial)

# defining the layers
def layers(x, W, b):
    return tf.nn.sigmoid(tf.matmul(x, W)+b)

sess = tf.Session()

LENGTH_GET = [80]
STEP = [[1,81]]
# LENGTH_GET = [8,16,32,48,64]
# STEP = [[1,9],[1,17],[1,33],[1,49],[1,65]]


# STEP = [[1, TIME + 1], [1, TIME + 1]]  # or Step = [10]

  # or Step = [10]

for size in range(len(LENGTH_GET)):

    # Size
    lx = LENGTH_GET[size]

    # Time
    ly = STEP[size][1] - STEP[size][0]

    # defining the model

    #first layer
    #weights and bias
    W_1 = weight_variable([lx * ly, hiddenunits1])
    b_1 = bias_variable([hiddenunits1])

    #Apply a sigmoid
    #x is input_data, y_ is the label
    x = tf.placeholder("float", shape=[None, lx * ly])
    y_ = tf.placeholder("float", shape=[None, numberlabels])

    O1 = layers(x, W_1, b_1)

    #second layer(output layer in this case)
    # W_2 = weight_variable([hiddenunits1, hiddenunits1])
    # b_2 = bias_variable([hiddenunits1])

    # O2 = tf.nn.relu(layers(O1, W_2, b_2))

    W_3 = weight_variable([hiddenunits1, numberlabels])
    b_3 = bias_variable([numberlabels])

    O3 = layers(O1, W_3, b_3)

    y_conv = O3

    #Train and Evaluate the Model

    # cost function to minimize (with L2 regularization)
    cross_entropy = tf.reduce_sum( -y_*tf.log(tf.clip_by_value(y_conv,1e-10,1.0))-(1.0-y_)*tf.log((tf.
```

```
                                                    clip_by_value(1-y_conv,1e-10,1.0))))  \
                 +lamb*(tf.nn.l2_loss(W_1)+tf.nn.l2_loss(W_3) )

#defining the optimizer
optimizer = tf.train.AdamOptimizer(learning_rate)  #0.0001 is learn_rate
train_step = optimizer.minimize(cross_entropy)

correct_prediction = tf.equal(tf.argmax(y_conv, 1), tf.argmax(y_, 1))
accuracy = tf.reduce_mean(tf.cast(correct_prediction, "float"))

#reading the data in the directory txt
mnist = input_data.read_data_sets(numberlabels,
                                  lx,
                                  ly,
                                  './data/',
                                  one_hot=True)

print(mnist)

print('test.images.shape', mnist.test.images.shape)
print('test.labels.shape', mnist.test.labels.shape)
print(
    "xxxxxxxxxxxxxxxxxxxxx Training START xxxxxxxxxxxxxxxxxxxxxxxxxxxxxxxxx",
    size)

sess.run(tf.global_variables_initializer())

# training
for i in range(1,trainstep+1):

    batch = mnist.train.next_batch(batch_size)

    if i % 2000 == 0:

        # batch_train = mnist.train.next_batch(batchsize_test)

        train_accuracy = sess.run(accuracy,
                                  feed_dict={
                                      x: batch[0],
                                      y_: batch[1]
                                  })
        print("step, train accuracy:", i, train_accuracy)

        # batch_test = mnist.test.next_batch(batchsize_test)

        # test_accuracy = sess.run(accuracy,
        #                           feed_dict={
        #                               x: batch_test[0],
        #                               y_: batch_test[1]
        #                           })
        # print("step, test accuracy:", i, test_accuracy)
    sess.run(train_step, feed_dict={x: batch[0], y_: batch[1]})

print(
    "xxxxxxxxxxxxxxxxxxxxx Training Done xxxxxxxxxxxxxxxxxxxxxxxxxxxxxxxxx"
)

print(
    "test accuracy",
    sess.run(accuracy,
             feed_dict={
                 x: mnist.test.images,
                 y_: mnist.test.labels
             }))

# saver = tf.train.Saver()
# save_path = saver.save(sess, "./model-saved.ckpt")
# print("Model saved in path: %s" % save_path)

print("xxxxxxxxxxxxxxxxxxxxx Plot Data xxxxxxxxxxxxxxxxxxxxxxxxxxxxxxxxx")
```

```
    #producing data to get the plots we like
    #output of neural net

    # plist = ptrain = [0, 0.05, 0.10000, 0.133, 0.166, 0.20000, 0.233, 0.266, 0.30000, 0.333,
    #                   0.366, 0.40000, 0.433, 0.466, 0.48, 0.50000, 0.533, 0.566, 0.58, 0.59,
    #                   0.60000,  0.61,   0.62,   0.63,   0.645,   0.66,   0.67,   0.68, 0.69,  0.70000,
    #                   0.715, 0.733, 0.766, 0.80000, 0.833, 0.866, 0.90000, 0.933, 0.966, 1.00000]
    plist = ptrain = [0.0375 + x*0.0025 for x in range(31)]
    ptest = plist
    Ntemp = len(
        plist)  # number of different temperatures used in the simulation

    samples_per_T = int(mnist.test.num_examples / Ntemp)

    f = open('./plot/' + 'fcnoutlx' + str(lx) + '_' + str(ly) + '.dat', 'w')
    ii = 0
    for i in range(Ntemp):
      av=0.0
      for j in range(samples_per_T):
            batch=(mnist.test.images[ii,:].reshape((1,lx*ly)),mnist.test.labels[ii,:].reshape((1,
                                                                        numberlabels)))
            res=sess.run(y_conv,feed_dict={x: batch[0], y_: batch[1]})
            av=av+res
            ii=ii+1
      av=av/samples_per_T
      print(plist[i],av[0,0],av[0,1])
      f.write(str(plist[i])+' '+str(av[0,0])+' '+str(av[0,1])+"\n")

    f.close()


    f = open('./plot/' + 'fcnacclx' + str(lx) + '_' + str(lx) + '.dat', 'w')
    ii = 0
    # accuracy vs temperature
    for i in range(Ntemp):
        batch = (mnist.test.images[ii * samples_per_T:ii * samples_per_T +
                                   samples_per_T, :].reshape(
                                       samples_per_T, lx * ly),
                 mnist.test.labels[ii * samples_per_T:ii * samples_per_T +
                                   samples_per_T, :].reshape(
                                       (samples_per_T, numberlabels)))
        train_accuracy = sess.run(accuracy,
                                  feed_dict={
                                      x: batch[0],
                                      y_: batch[1]
                                  })
        ii = ii + 1
        f.write(str(ptest[i]) + ' ' + str(train_accuracy) + "\n")

    f.close()

end = time.time()
print('Running time: %s Seconds'%(end-start))
```

The autoencoder algorithm

The PCPD' autoencoder algorithm is as follows:

```
import tensorflow as tf
import input_data
import sys
import numpy as np
import seaborn as sns
import matplotlib.pyplot as plt

numberlabels = 2
hiddenunits = [2*512,64, 2, 64,512*2]

lamb = 0.001  # regularization parameter
```

```
batchsize_test = 1000
learning_rate = 0.0001
batch_size = 32
trainstep = (2500 * 10 // batch_size)*20
kernel_size = 3
kernel_size_1 = 3
kernel_size_2 = 3
def weight_variable(shape):
    initial = tf.truncated_normal(
        shape, stddev=0.01)
    return tf.Variable(initial)

def bias_variable(shape):
    initial = tf.constant(
        0.01, shape=shape)   #shape=[2]      [[0.01 0.01] [0.01 0.01]]
    return tf.Variable(initial)

# defining the layers
def layers(x, W, b):
    return tf.nn.sigmoid(tf.matmul(x, W)+b)

def layers_R(x, W, b):
    return tf.nn.relu(tf.matmul(x, W)+b)

def layers_N(x, W, b):
    return tf.matmul(x, W)+b

def conv2d(x,W,b):
    return tf.nn.relu(tf.nn.conv2d(x,W,[1,1,1,1],padding='SAME')+b)

def conv2d_S(x,W,b):
    return tf.nn.sigmoid(tf.nn.conv2d(x,W,[1,1,1,1],padding='SAME')+b)

def max_pool_2(x):
    return tf.nn.max_pool(x,[1,2,2,1],[1,2,2,1],padding='SAME')

sess = tf.Session()

LENGTH_GET = [40]
STEP = [[1,41]]
# LENGTH_GET = [8,16,32,48,64]
# STEP = [[1,9],[1,17],[1,33],[1,49],[1,65]]

for size in range(len(LENGTH_GET)):
    # Size
    lx = LENGTH_GET[size]
    # Time
    ly = STEP[size][1] - STEP[size][0]
    # defining the model
        #x is input_data, y_ is the label
    x = tf.placeholder("float", shape=[None, lx * ly])
    y_ = tf.placeholder("float", shape=[None, lx * ly])

    xinput_re = tf.reshape(x,[-1,lx,ly,1])
    # encoder
    W_1 = weight_variable([kernel_size_1,kernel_size_2,1,16])
    b_1 = bias_variable([16])
    O1 = conv2d_S(xinput_re, W_1, b_1)   #lx*lx
    # 100 > 50
    W_2 = weight_variable([kernel_size_1, kernel_size_2, 16, 8])
    b_2 = bias_variable([8])
    O2 = conv2d_S(max_pool_2(O1), W_2, b_2)#(lx//2 ) *(lx//2)

    W_21 = weight_variable([kernel_size_1, kernel_size_2, 8, 8])
    b_21 = bias_variable([8])
    O21 = conv2d_S(max_pool_2(O2), W_21, b_21) #(lx//4 ) *(lx//4)
    O2_re = tf.reshape(max_pool_2(O21),[-1,(lx//8 )*(ly//8)*8])   #(lx//8 ) *(lx//8)
    # 50 > 2
    W_3 = weight_variable([(lx//8 )*(ly//8)*8, 1])
    b_3 = bias_variable([1])
```

```python
O3 = layers_N(O2_re, W_3, b_3)
# decoder
W_4 = weight_variable([1, (lx//8 )*(ly//8)*8])
b_4 = bias_variable([(lx//8 )*(ly//8)*8])
O4 = layers(O3, W_4, b_4)
O4_re = tf.reshape(O4,[-1,lx//8,ly//8,8])
# 50 > 100
W_5 = weight_variable([kernel_size_1,kernel_size_2,8, 8])
b_5 = bias_variable([8])
O5 = conv2d_S(tf.image.resize_images(O4_re,(lx//4,ly//4),method=1), W_5, b_5)

W_51 = weight_variable([kernel_size_1,kernel_size_2,8, 16])
b_51 = bias_variable([16])
O51 = conv2d_S(tf.image.resize_images(O4_re,(lx//2,ly//2),method=1), W_51, b_51)
# 100 > lx*ly
W_6 = weight_variable([kernel_size_1,kernel_size_2,16,16])
b_6 = bias_variable([16])
O6 = conv2d_S(tf.image.resize_images(O51,(lx,ly),method=1), W_6, b_6)

W_7 = weight_variable([kernel_size_1,kernel_size_2,16,1])
b_7 = bias_variable([1])
O7 = conv2d_S(O6, W_7, b_7)

y_conv = tf.reshape(O7,[-1,lx*ly])

#Train and Evaluate the Model
# cost function to minimize (with L2 regularization)
cross_entropy = tf.reduce_mean( -y_*tf.log(tf.clip_by_value(y_conv,1e-10,1.0))-(1.0-y_)*tf.log((tf.
                                                    clip_by_value(1-y_conv,1e-10,1.0))))
#defining the optimizer
optimizer = tf.train.AdamOptimizer(learning_rate)  #0.0001 is learn_rate
train_step = optimizer.minimize(cross_entropy)


#reading the data in the directory txt
mnist = input_data.read_data_sets(numberlabels,
                                  lx,
                                  ly,
                                  './data/',
                                  one_hot=True)

print(mnist)

print('test.images.shape', mnist.test.images.shape)
print('test.labels.shape', mnist.test.labels.shape)
print(
    "xxxxxxxxxxxxxxxxxxxxxx Training START xxxxxxxxxxxxxxxxxxxxxxxxxxxxxxxxx",
    size)

sess.run(tf.global_variables_initializer())

# training
for i in range(1,trainstep+1):

    batch = mnist.train.next_batch(batch_size)

    if i % 2000 == 0:

        # batch_train = mnist.train.next_batch(batchsize_test)

        train_loss = sess.run(cross_entropy,
                              feed_dict={
                                  x: batch[0],
                                  y_: batch[0]
                              })
        print("step, train loss:", i, train_loss)



    sess.run(train_step, feed_dict={x: batch[0], y_: batch[0]})
```

```
    print(
        "xxxxxxxxxxxxxxxxxxxxx Training Done xxxxxxxxxxxxxxxxxxxxxxxxxxxxxxxxx"
    )

    print(
        "test loss",
        sess.run(cross_entropy,
                 feed_dict={
                     x: mnist.test.images,
                     y_: mnist.test.images
                 }))

    print("xxxxxxxxxxxxxxxxxxxxx Plot Data xxxxxxxxxxxxxxxxxxxxxxxxxxxxxxxxx")

    #producing data to get the plots we like
    #output of neural net
    # plist = ptrain = [0.1 + x*0.1 for x in range(10)]
    # plist = ptrain = [0.1,0.2,0.3,0.4,0.5,0.6,0.7,0.8,0.9,1.0]
    plist = ptrain = [0.0 + x*0.01 for x in range(31)]
    # plist = ptrain = [0.0 + x*0.025 for x in range(41)]
    # plist = ptrain = [0.0 + x*0.005 for x in range(41)]
    # plist = ptrain = [0.1, 0.6447, 0.9]
    ptest = plist
    Ntemp = len(plist)  # number of different temperatures used in the simulation
    print(Ntemp)
    samples_per_T = int(mnist.test.num_examples / Ntemp)

    f = open('./plot/' + 'nnoutlxalldensityd02' + str(lx) + '_' + str(ly) + '.dat', 'w')
    ii = 0
    av_T =[]
    av_x_ALL= []
    av_y_ALL= []
    for i in range(Ntemp):
        # av_z = []
        av=0.0
        for j in range(samples_per_T):

            res=sess.run(O3,feed_dict={x: [mnist.test.images[ii]]})
            # print(res)
            # av.append(res[0])
            av=av+res
            ii +=1
            # if ii == 25000:
            #     # print(mnist.train.images[ii,:])
            #     plt.imshow(mnist.train.images[ii,:].reshape((lx,ly)))
            #     plt.show()
            #     plt.imshow(sess.run(O7,feed_dict={x: batch[0], y_: batch[0]}).reshape((lx,ly)))
            #     plt.show()
        av=av/samples_per_T
        # av_z_ALL.append(av_z)
        print(av)
    # for i in range(len(plist)):
        plt.scatter(plist[i],av[0][0],label="{}".format(plist[i]))
        f.write(str(plist[i])+' '+str(av[0,0])+' '+"\n")
    plt.xlabel('${p}$',fontsize=20)
    plt.ylabel('${h^*}$',fontsize=20)
    # plt.figure(figsize=(8,6))
    # plt.title('A single hidden neuron activation as a function of ${p}$')
    plt.savefig('ae_pc_alldensity.pdf')
    plt.show()
```

The PCA algorithm

The PCPD's principal components analysis(PCA) algorithm is as follows:

```
import json
import numpy as np
import sys
from sklearn.decomposition import PCA
```

```
from sklearn import preprocessing
from sklearn.utils.extmath import svd_flip
import matplotlib.pyplot as plt
from matplotlib.patches import Ellipse
from matplotlib import rc,rcParams
import matplotlib
import random
import time
from matplotlib.ticker import MultipleLocator, FormatStrFormatter
start=time.time()

# font = {'size':30}
# matplotlib.rc('font', **font)

# -------------------------------------------------------
def Update(V_pro, dl, dr, ann, cl):
    L = np.shape(V_pro)[0]
    V_aft = np.copy(V_pro)
    for i in range(L):
        h = (i - 1 + L) % L
        j = (i + 1) % L
        k = (i + 2) % L
        l = (i + 3) % L

        prob = random.random()
        if prob<dl: #diff to right
            if V_pro[j] == 1 and  V_pro[k] ==0:
                V_aft[j] =0
                V_aft[k] =1

        elif prob<dr: #diff to left
            if V_pro[j] == 1 and  V_pro[i] ==0:
                V_aft[j] =0
                V_aft[i] =1

        elif prob < ann:
            if V_pro[j] ==1 and V_pro[k] ==1:
                V_aft[j] =0
                V_aft[k] =0

        elif prob < cl: #create at right
            if V_pro[j] ==1 and V_pro[k] ==1 and V_pro[l] ==0:
                V_aft[l] =1

        else: #create at left
            if V_pro[i] ==1 and V_pro[j] ==1 and V_pro[h] ==0:
                V_aft[h] =1
    return V_aft

run_time = 100
d = 0.1
pc = 0.10688
dl = d/2
dr = d
# ptrain = [0.1, 0.6447, 0.9]
ptrain = [0.0 + x*0.01 for x in range(31)]
# ptrain = [0.0 + x*0.01 for x in range(31)]
ptest = ptrain
plist = ptest
z = 1.

LENGTH = 40
# TIME = np.math.ceil(LENGTH**z)
# print(TIME)
TIME = LENGTH-1
print(TIME)

a_test = []
for p in ptest:
    ann = dr + p*(1-dr)
```

```
    cl = ann + (1-p)*(1-dr)/2
    for icyc in range(0, run_time):
        # vector = []
        vector_LT = []
        V_pro = np.ones(LENGTH)
        vector_LT.extend(V_pro)
        for i in range(TIME):
            V_pro = Update(V_pro, dl, dr, ann, cl)
            vector_LT.extend(V_pro)
        a_test.append(vector_LT)

aa_test = []
for p in ptest:
    for icyc in range(0, run_time):
        array = []
        array = p
        aa_test.append(array)
# print(aa_test)

X = np.array(a_test)
print(X.shape)
y = np.array(aa_test)
print(y)
print(y.shape)
# target_names = iris.target_names
# pca = PCA(n_components=2)
pca = PCA(n_components = 10, svd_solver='full')
# print(pca)
pca.fit(X)
# print(pca.fit(X))
X_reduction = pca.transform(X)
namda = pca.explained_variance_ratio_
print(namda)
print(namda.shape)
# print(pca.singular_values_)

# print(X_reduction)
# print(X_reduction.shape)
# print (pca.n_components_)
# print(X_reduction[100,0])

#--------------------------------------------------------------------------
# To make plots pretty
golden_size = lambda width: (width, 2. * width / (1 + np.sqrt(5)))
font1 = {'family': 'Times New Roman',
         'weight': 'normal',
         'size': 60,
         }
plt.rc('font',**{'size':60})
# rc('text',usetex=True)
# rcParams.update({'font.size': 16})
fig, ax = plt.subplots(1,figsize=golden_size(26))
order = [1 + x*1 for x in range(10)]
print(order)
ax.plot(order[0:], namda[0:], color='red')
sc = ax.scatter(order[0:], namda[0:], s=60,marker = "x", color='blue')
ax.set_xlabel(u'${\ell}$',fontsize=60)
ax.set_ylabel(u'$ \~{\lambda}_{\ell} $',fontsize=60)
y=[pow(10,i) for i in range(-3,0)]
plt.yscale('log')
ax.set_ylim(10**-3, 10**0)
plt.xlim(0,10.5)
# plt.ylim(-pow(10,-0.8),pow(10,0))
ax.tick_params(axis='both',which='both',direction='in',top=True,right=True,labeltop=False,labelright =
                                                    False)
plt.tick_params(which='major',width=4,length=32)
plt.tick_params(which='minor',width=4,length=16)

xmajorLocator = MultipleLocator(2)
xmajorFormatter = FormatStrFormatter('%.0f')
```

```
xminorLocator    = MultipleLocator(1)
# ymajorLocator = MultipleLocator(0.5)
# ymajorFormatter = FormatStrFormatter('%.1f')
# yminorLocator   = MultipleLocator(0.1)

ax.xaxis.set_major_locator(xmajorLocator)
ax.xaxis.set_major_formatter(xmajorFormatter)
ax.xaxis.set_minor_locator(xminorLocator)

# ax.yaxis.set_major_locator(ymajorLocator)
# ax.yaxis.set_major_formatter(ymajorFormatter)
# ax.yaxis.set_minor_locator(yminorLocator)

# plt.colorbar(sc, label='$0.25\\times$Temperature')
# plt.colorbar(sc)
# plt.legend(sc)
plt.savefig('pcpd1d_varianceratio.pdf')

end=time.time()
print('Running time: %s Seconds'%(end-start))

plt.show()
```

PCPD's Monte Carlo simulations

PCPD' MC simulations with C++ code is as follows:

```
// compile with: g++ -std=c++11 -O3 pcpd1dml.cpp -o pcpd1dml
// usage: ./pcpd1dml D p L T poccup RUN
///Parameters:
// D: diffusion rate; p: contral parameter; L: system size;
// T: simulation time steps; poccup: initial occupation probability;
// RUN: number of runs
#include <iostream>
#include <fstream>
#include <string>
#include <numeric>
#include <cmath>
#include <sys/time.h>
#include <ctime>
#include <climits>
#include <cstdlib>

using namespace std;

// time seed
struct timeval time_now{};
time_t msecs_time;

// lateral size, simulation time steps, number of runs, number of thread for each job
int L, T, TT, RUN;

// initial occupation probability
float poccup;

/* rates */
float d, p;

/* propensities */
float dl, dr, ann, cl /*,cr*/;
```

```
/////////////////// - xoshiro256** -//////////////////////
static inline uint64_t rotl(const uint64_t x, int k) {
        return (x << k) | (x >> (64 - k));
}

uint64_t next(uint64_t (&st)[4]) {
        const uint64_t result_starstar = rotl(st[1] * 5, 7) * 9;

        const uint64_t t = st[1] << 17;

        st[2] ^= st[0];
        st[3] ^= st[1];
        st[1] ^= st[2];
        st[0] ^= st[3];

        st[2] ^= t;

        st[3] = rotl(st[3], 45);

        return result_starstar;
}

double get_rand(uint64_t (&st)[4]) {
        return static_cast<double>(next(st)) / UINT64_MAX;
}
//////////////////////////////////////////////////


void Move(bool& sitei, bool& sitej) {
        if (sitei && !sitej) { sitei = 0; sitej = 1;}
}

void Annihilate(bool& sitei, bool& sitej) {
        if (sitei && sitej) { sitei = 0; sitej = 0;}
}

void Create(bool& sitei, bool& sitej, bool& sitek) {
        if (sitei && sitej && !sitek) { sitek = 1; }
}

void Update(bool* s, uint64_t (&st)[4], int i, int L)
{
        int h, /*i*/ j, k, l;
        h = (i - 1 + L) % L;
        j = (i + 1) % L;
        k = (i + 2) % L;
        l = (i + 3) % L;

        double r = get_rand(st);
        if (r<dl) Move(s[j], s[k]); // diff to right
        else if (r<dr) Move(s[j], s[i]); //diff to left
        else if (r<ann) Annihilate(s[j], s[k]);
        else if (r<cl) Create(s[j], s[k], s[l]); //create at right
```

```
        else Create(s[i], s[j], s[h]); // create at left
}

void doPCPD(bool* s, uint64_t (&st)[4], int L) {
        for (int i = 0; i < L; i++) Update(s, st, next(st)%L, L);
        //for (int i = 1; i < L; i+=2) Update(i);
        //for (int i = 0; i < L-1; i+=2) Update(i);
}

void do_it(int L, int RUN, float poccup, double* rho, int* surv, uint64_t (&st)[4])
{
   bool s[L];
   for (int run = 0; run < RUN; run++) {
     if (poccup == 1) fill_n(s, L, 1);
     else {
         for (int i = 0; i < L; i++) {
             if (get_rand(st) < poccup) s[i] = 1;
             else s[i] = 0;
         }
     }
     for (int y = 0; y < T; y++) {
       if (y%5==0) {
           int NA = accumulate(s, s+L, 0);
           rho[y/5] += (double)(NA)/L;
           if (NA > 1) surv[y/5] += 1;
       }
       doPCPD(s, st, L);
       }
  }
}


int main(int argc, char *argv[]){
   gettimeofday(&time_now, nullptr);
   time_t msecs_time = (time_now.tv_sec * 10000) + (time_now.tv_usec / 100); // time since ep

   static uint64_t st[4] = { 0x180ec6d33cfd0aba*(uint64_t)(msecs_time), 0xd5a61266f0c9392c*(u

   ///////////////// - rates -//////////////
   d = strtod(argv[1], NULL);
   p = strtod(argv[2], NULL);

   dl = d/2; dr = d;
   ann = dr + p*(1-dr);
   cl = ann + (1-p)*(1-dr)/2;
   //////////////////////////////////////

   L = stoi(argv[3]);                  // lateral size
   T = stoi(argv[4]);                  // simulation time steps
   TT = T/5;
   poccup = strtod(argv[5], NULL);     // initial occupation probability
   RUN = stoi(argv[6]);                // number of runs

   double rho[TT] = {0};
   int surv[TT] = {0};
```

```
    string filename, D, Pval, Lval, Tval, poccupval, RUNval, time_stamp;
    D = argv[1]; Pval = argv[2]; Lval = argv[3]; Tval = argv[4];
    poccupval = argv[5]; RUNval = argv[6];
    time_stamp = to_string(msecs_time);
    filename = "PCPD1d_"+D+"_"+Pval+"_"+Lval+"_"+Tval+"_"+poccupval+"_"+RUNval+"_"+time_stamp+

    ofstream outfile;
    outfile.open(filename.c_str());

    do_it(L, RUN, poccup, rho, surv, st);

    for (int time=0; time<T/5; time++) {
        outfile << 5*time << "\t" << rho[time]/RUN << "\t" << (double)(surv[time])/RUN << endl
    }
    outfile.close();

    return 0;
}
```
